# Supplementary material for: The virome of Drosophila suzukii, an invasive pest of soft fruit
Source: Virus Evol. 2018 Mar 29;4(1):vey009. doi: 10.1093/ve/vey009 (PMC5888908; doi:10.1093/ve/vey009)
Supplement: Supplementary Data [file vey009_supp.zip › S1_figure_coverage.pdf]

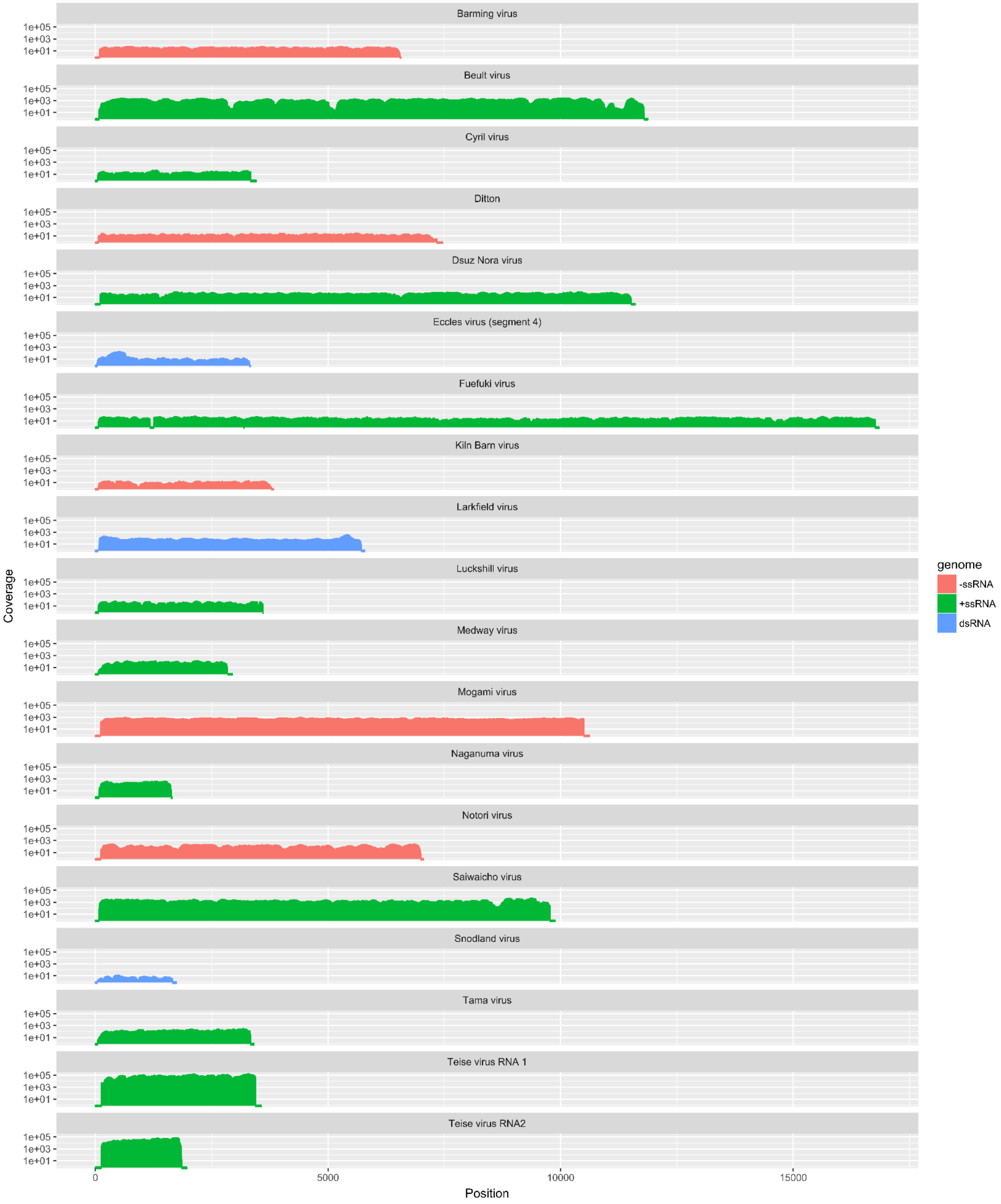

S1_figure. Coverage depth of each nucleotide in newly described virus genomes. Raw 150nt forward reads from pools where reference virus was most common are mapped to putative virus genomes using Bowtie2 (2.3.0).
